# Mesenchymal Stem Cells Empowering Tendon Regenerative Therapies

**DOI:** 10.3390/ijms20123002

**Published:** 2019-06-19

**Authors:** Raquel Costa-Almeida, Isabel Calejo, Manuela E. Gomes

**Affiliations:** 1 3B’s Research Group, I3Bs—Research Institute on Biomaterials, Biodegradables and Biomimetics, University of Minho, Headquarters of the European Institute of Excellence on Tissue Engineering and Regenerative Medicine, AvePark, Parque de Ciência e Tecnologia, Zona Industrial da Gandra, 4805-017 Barco, Guimarães, Portugal; raquel.almeida@i3bs.uminho.pt (R.C.-A.); isabel.calejo@i3bs.uminho.pt (I.C.); 2 ICVS/3B’s—PT Government Associate Laboratory, 4805-017 Barco, Guimarães, Portugal; 3 The Discoveries Centre for Regenerative and Precision Medicine, Headquarters at University of Minho, Avepark, 4805-017 Barco, Guimarães, Portugal

**Keywords:** adipose-derived stem cells, bone marrow derived stem cells, cellular communication, Extracellular matrix, tendon healing, tendon stem/progenitor cells, tenogenesis

## Abstract

Tendon tissues have limited healing capacity. The incidence of tendon injuries and the unsatisfactory functional outcomes of tendon repair are driving the search for alternative therapeutic approaches envisioning tendon regeneration. Cellular therapies aim at delivering adequate, regeneration-competent cell types to the injured tendon and toward ultimately promoting its reconstruction and recovery of functionality. Mesenchymal stem cells (MSCs) either obtained from tendons or from non-tendon sources, like bone marrow (BM-MSCs) or adipose tissue (ASCs), have been receiving increasing attention over the years toward enhancing tendon healing. Evidences from in vitro and in vivo studies suggest MSCs can contribute to accelerate and improve the quality of tendon healing. Nonetheless, the exact mechanisms underlying these repair events are yet to be fully elucidated. This review provides an overview of the main challenges in the field of cell-based regenerative therapies, discussing the role of MSCs in boosting tendon regeneration, particularly through their capacity to enhance the tenogenic properties of tendon resident cells.

## 1. Introduction

Cells are Nature’s tissue engineers, intervening, through a spatiotemporal coordinated manner, in tissue homeostasis, disease development, tissue repair/healing, and ultimately regeneration. Within the Metazoa kingdom, regenerative abilities are unequally distributed [1]. Regeneration refers to post-natal restoration of a lost body part/structure and can occur through a variety of developmental mechanisms to originate structures that closely resemble the original prior to injury [1]. Replicating tissue development in mammals is a dramatic challenge, owing to the limited knowledge about signals involved in the initiation of regenerative processes. Upon injury, tissue healing typically occurs through a continuous process comprising three interdependent and overlapping stages: an initial inflammatory response, followed by a fibroblastic and/or proliferative stage and, finally, a prolonged remodeling phase [2,3]. In several cases, tissue healing is often associated with a pro-fibrotic scenario that leads to scar formation, rather than normal tissue regeneration [4].

Regenerative medicine, including tissue engineering, envisions the generation of adequate therapeutic strategies to restore the functionality of an injured tissue or organ. Central to these approaches, cells are key players in orchestrating regenerative responses and thus, are the main targets of any strategy envisioning tissue regeneration. Thus, despite the fact that different approaches can be considered, all rely on controlling to some extent cellular responses, which implies knowing the biology of the target tissue. Within the human body, tendons are highly prone to fibrotic healing through excessive and disorganized deposition of the extracellular matrix (ECM) [3]. This repair process is differentially regulated between fetal and adult tendon tissues [5,6,7].

Over the years, regenerative tendon approaches have been exploiting (i) cellular therapies [8,9]; (ii) injections of platelet rich-hemoderivatives (PRHd), like platelet-rich plasma (PRP) [10,11]; (iii) gene therapy; [12] and (iv) biomaterial-based strategies [13,14] aiming at promoting tissue regeneration and inhibiting peritendinous adhesions formation [3]. Given the biomechanical role of these musculoskeletal tissues, biomaterial-based strategies envision the development of tissue substitutes. However, cells have a primary role in maintaining tendon ECM dynamics and normal function and, thus, cell-based strategies are gaining attention, mainly through the combination of cells and biomaterials. In particular, mesenchymal stem cells (MSCs) have been extensively explored for musculoskeletal tissue engineering and regeneration, including for tendon applications [15]. In this review, we address the use of MSCs from tendon and non-tendon origin and their contributions to tendon healing. Firstly, the need for tendon regenerative therapies is tackled and the main characteristics of tendon cell populations and cellular niche are briefly addressed as the key environment providing insights on relevant signals to potentially direct tenogenic differentiation of stem cells. Finally, the use of cellular therapies envisioning a shift from pro-fibrotic tissue repair to tissue regeneration is discussed.

## 2. Tendon Pathophysiology

### 2.1. A Snapshot on Tendon Cellular Niche

**Figure 1:**
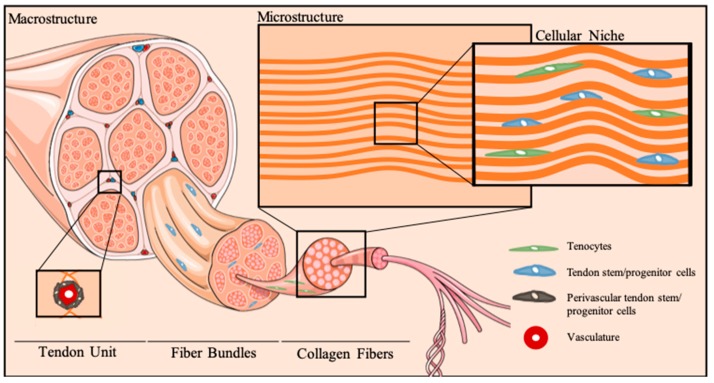
Tendon cellular niche. Schematic representation of tendon hierarchical organization and micro-to-macro structural architecture of the cellular niche. Tenocytes reside between anisotropically-aligned collagen fibers. Multipotent stem cell populations, termed tendon stem/progenitor cells, can be found in several stem cell niches in tendons, particularly the perivascular niche, as well as other tendon areas, as the epi-, peri- and endotenon.

Tendons are highly organized connective tissues comprising bottom-up assemblies of collagen molecules (Figure 1). This hierarchical arrangement of the extracellular matrix (ECM) guarantees the unique biomechanical performance of tendons, which is responsible for assuring effective loading transmission between muscles and bones and consequent body movements. Tendon ECM is comprised mainly of collagen type I, followed by collagen type III, as well as proteoglycans (decorin, fibromodulin, biglycan, lumican, aggrecan, and versican) and glycosaminoglycans (dermatan sulfate and chondroitin sulfate), as reviewed elsewhere [16,17].

Tendons are relatively hypocellular tissues. Nonetheless, tendon cell populations assure ECM dynamics during tissue homeostasis. As a result of the stretching mechanical forces, tendon cells exhibit a characteristic elongated, spindle-shape morphology. Traditionally, tendons were believed to comprise a homogenous population of tenocytes, which are differentiated cells expressing common tendon markers, such as scleraxis (Scx), a basic loop-helix-loop transcription factor, tenomodulin (Tnmd), a transmembrane glycoprotein, as well as the tendon ECM component collagen type I (Col1) [18,19]. Nonetheless, tendon cell populations have been shown to include tendon stem/progenitor cells (TSPCs) [20], containing cells that fulfill the universal criteria of mesenchymal stem cells (MSCs)—clonogenecity, multipotency and self-renewal. Despite the knowledge gap on tendon biology, it is well recognized that both differentiated and TSPCs reside within a unique microenvironment of biophysical and biochemical signals [17,21]. Particularly, stretching mechanical forces and ECM topography are dominating factors governing tissue function and cellular processes [22]. Furthermore, a balance of biochemical factors tightly regulates the orchestration of biological processes in tendon. Specifically, tendon healing involves numerous cytokines (e.g., interleukin (IL)-6, IL-1β) and growth factors (e.g., basic fibroblast growth factor (bFGF), transforming growth factor (TGF)-β, insulin-like growth factor (IGF)-1, platelet-derived growth factor (PDGF) and bone morphogenetic proteins (BMP)-12, -13, and -14), which are released in a temporally- and spatially-controlled manner [23,24,25]. Notwithstanding, it is worth mentioning that inflammatory cells play a crucial role in tendinopathies and tendon healing [3,26], intervening in the dynamics of tendon cellular niche.

Although the cellular and molecular mechanisms intervening in tendon development, homeostasis and repair are still to be fully uncovered, it is evident that an interplay between biophysical and biochemical signals coordinates cellular function and impacts tenogenic differentiation of stem cells.

### 2.2. Tendinopathies: A Painful Global Burden and the Need for Novel Therapies

Musculoskeletal conditions are the second largest contributor to disability, affecting people across the life-course worldwide. The Global Burden of Disease (GBD) study has estimated that 20–33% of people globally live with a painful musculoskeletal condition [27,28]. Among these, it is estimated that over 30 million human tendon/ligament-related procedures are taking place annually worldwide, representing an associated expenditure of over €150 billion euros in the USA and EU [8,29]. These treatments pose a considerable financial burden on healthcare systems and are expected to increase as a consequence of increased life expectancy and contemporary sedentary lifestyles [30], as well as of the rising prevalence of overweight, obesity and metabolic diseases [31].

Tendinopathies are commonly divided into two major classes: acute and chronic (degenerative) damage [32]. Acute tendinopathies are associated with traumatic damage of previously healthy tissue, whereas chronic tendinopathies (>3 months) are associated with overuse tendon injuries and frequently involve an unresolved inflammatory scenario and impaired performance [10]. Current therapeutic strategies frequently resort to surgical repair through suturing of the damaged tissues or the application of auto- or allo-grafts, whenever conservative treatments fail or are not appropriate. Furthermore, various artificial grafts/scaffolds for tendon/ligament substitution are commercially available, including both biological and synthetic scaffolds. However, a satisfactory functional outcome is still to be achieved given the drawbacks associated to these strategies, as reviewed elsewhere [33].

In general, the quality of repaired tendon tissue rarely returns to pre-injury levels, leading to high re-injury risk and the onset of degenerative changes related to the development of chronic tendinopathies [34,35]. Given the limited success of current clinical treatments, the management of tendon injuries and disorders requires the development of more effective strategies toward ultimately regenerating the injured tissue and recovering functionality.

## 3. Cell-Based Therapies for Tendon Applications

Cellular therapies envision the delivery of regeneration-competent cell populations, i.e., cells that are able to repopulate the injured tissue and/or of empowering resident cell populations with the ultimate goal of promoting tissue reconstruction and functional recovery. Tendon healing frequently encompasses the differentiation of resident TSPCs toward a phenotype resembling that of activated fibroblasts, leading to scar tissue formation, exacerbated inflammatory response and impaired functionality [7,36]. In this sense, the use of MSCs envisions a modulation of the inflammatory environment targeting a potential shift from pro-inflammatory and pro-fibrotic to pro-regenerative cellular responses, leading to a reduced infiltration of inflammatory cells and ordered deposition of ECM components [3,37]. Given the hypocellular nature of tendons, the application of cell-based therapies is quite intriguing and several challenges need to be addressed, as discussed in detail below.

### 3.1. General Considerations

MSCs have been long described to possess multilineage differentiation ability in vitro and have been widely used for applications in Tissue Engineering and Regenerative Medicine, including in the field of musculoskeletal repair [15]. Notwithstanding, the functional role of MSCs in regenerative therapies is being increasingly discussed and evidences exist that the therapeutic effects of these cells could be attributed mainly to their function of empowering resident cells [38,39,40], raising controversy over the terminology [41].

Adult stem cells of mesenchymal origin hold great interest in the field of tendon tissue engineering and regeneration as promising alternatives to the scarce population of tendon cells. Herein, we will discuss the main outcomes of strategies using MSCs from different sources for tendon repair. Nonetheless, pluripotent stem cells have also been explored in the field, namely embryonic stem cells and induced pluripotent stem cells (iPSCs) [42,43]. In particular, iPSCs can be easily obtained in an autologous setting, which facilitates clinical translation. The combination of iPSCs-derived MSCs with anisotropically-aligned fibrous biomaterial has proven beneficial for collagen production during tendon healing [44]. Notwithstanding, the use of iPSCs still faces several challenges and direct reprogramming of somatic cells has been attracting increasing interest, although very limited in the field of tendon applications owing to the lack of specific tendon markers. Alternatively, fibroblasts from different sources, including not only tendons and ligaments, but also skin, have been applied as well [45].

### 3.2. Main Challenges

Key issues to be considered when attempting to translate bioengineering strategies to tendon therapeutic approaches include (i) the lack of standardized isolation protocols, (ii) the inexistence of tendon-specific molecular markers and (iii) the definition of adequate differentiation protocols. Consequently, given the lack of well-established/standardized differentiation protocols and characterization markers for tenogenic differentiation, the selection of an ideal cell source represents a major challenge while trying to establish an effective cellular therapy.

• Isolation protocols

Several problems remain unsolved concerning the isolation of tendon cell populations. Isolation of TSPCs and tenocytes still lacks the tune of enrichment and selection approaches. Tendon cells were initially isolated following collagenase digestion of tendon explants [46,47]. However, over the years, several isolation methods have been described for obtaining TSPCs, also commonly termed tendon-derived stem cells (TDSCs), lacking a consensus between the type of digestion mix used, type of digestion mix used and time of digestion. Additionally, difficulties in characterizing tendon cells (as discussed below) have been limiting the definition of an optimal protocol. Table 1 presents examples of isolation protocols and main outcomes. Over the years, several protocols with slight variations have been described, but differences among cell characterization techniques and markers used have been challenging the field. Moreover, it is most likely that a mixed cell population is normally obtained, as it presents heterogeneous characteristics in terms of differentiation, clonogenecity and self-renewal capability [48]. Various studies assessed the multi-differentiation capacity of isolated cells, as well as the expression of tenogenic markers upon cell isolation. Nonetheless, TSPCs and tenocytes are commonly isolated in the same studies, and cell morphology is frequently used as the only aspect to identify and distinguish between cell populations. Tenocytes were traditionally described as small and fusiform cells [46,47], but over the years, different phenotypes have been reported based on nuclei morphology. Particularly, “resting” tenocytes within the tensional region of tendons exhibit spindle-shaped nuclei; “active” tenocytes possess a more elongated nucleus and “chondrocytic” tenocytes, which reside in compressed regions of tendons have round nuclei [49,50,51,52]. The first two types reside between collagen fibers and exhibit several cytoplasmic extensions allowing cell-cell communication through gap junctions, namely connexin (Cx)-32 and Cx-43 [53]. In turn, TSPCs are frequently described as large polygonal and star shaped cells, exhibiting round nuclei with prominent nucleoli, but acquiring a fibrobblast-like shape over passaging [20,49,54]. Therefore, some controversy may arise regarding the identity of these cell populations.

• Molecular signature

Tendon cells are highly-specialized fibroblasts, being the main producers of tendon ECM components. Given their mesenchymal origin and the limitations of characterizing fibroblasts and distinguishing them from MSCs [45], the definition of an accurate molecular signature is still far from being achieved. Hence, the inexistence of molecular markers to discriminate between tendon cell populations, as well as to characterize every discrete step of cell lineage specification makes the purification and differentiation of TDSCs and TSPCs very complicated. In comparison to other MSCs, tendon stem cells are known to share common surface markers, to express identical genes and to respond in a similar manner to growth factor stimulation, however, it has been recognized that the expression profiles, although very alike, are not identical [20] and are species-dependent.

Strikingly, several factors such as age, donor variability, tendon type, and anatomic location, alongside with culture conditions, have also been reported to influence markers expression in these cell populations [20].

• Differentiation protocols

The establishment of adequate differentiation methods relies on the optimization of inductive protocols to commit stem cells toward a certain lineage. In opposition to chondrogenic and osteogenic differentiation, there is no standard induction protocol for tenogenesis. Generally, different growth factors can be added to culture medium to induce the desired phenotype, as these biomolecules are powerful tools in regulating biological responses. These regulatory effects have been demonstrated, for instance, after culturing human adipose-derived stem cells (ASCs) or human amniotic fluid stem cells (AFSCs) in the presence of growth factors associated with tendon development and healing, namely endothelial growth factor (EGF), PDGF-BB, bFGF, and TGF- β1 [68]. An upregulation of tendon-related genes demonstrated the potential use of biochemical molecules to induce cellular commitment toward the tenogenic lineage, with differential effects over the two cell types. Indeed, EGF and bFGF exerted more pronounced effects over AFSCs, whereas ASCs responded more evidently to EGF and PDGF-BB [68]. Furthermore, medium supplementation with TGF-β3 showed a potent regulatory influence of this growth factor over the temporal profile of tenogenic genes in both ASCs and bone marrow-derived mesenchymal stem cells (BM-MSCs) cultures [69]. In addition, studies investigating the effect of different combinations of growth factors in either 2D or 3D culture [70], as well as further combining with mechanical stimulation [71], are opening new avenues toward differentiating MSCs into the tenogenic lineage. Strategies targeting tenogenic differentiation through the addition of growth factors or by mechanical stimulation have been recently reviewed [72]. However, an ideal medium formulation is not yet available.

Even though several attempts have been made over the years to commit stem cells towards the tenogenic lineage, the identification of several major tenogenic biomarkers is of major importance. As the molecular signature of tendon cells is still to be uncovered, it is still difficult to clearly understand and clarify the differentiation process occurring in several cultures and in the proper tissue, posing a strong challenge to the development of effective cell-based therapies.

### 3.3. Mesenchymal Stem Cells for Tendon Regenerative Therapies

MSCs can be applied in either autologous or allogeneic settings and can be obtained from tendon or non-tendon tissues. In the following sections, the contribution of MSCs from different sources to tendon healing, and ultimately regeneration, is discussed.

#### 3.3.1. Tendon Stem Cells

Tendon stem cells are frequently obtained as a heterogeneous population of stem and progenitor cells, as highlighted before. The purity of tendon cell populations is highly debatable, but these cells are believed to hold potential for improving tendon repair mechanisms. Autologous tenocyte implantation is currently under clinical studies (Phase 2-3 clinical trial, NCT01343836), but it is not clear whether the transplanted cells include only differentiated cells or a mixed population comprising also TDSCs or TSPCs.

Tendon stem cells represent 1–4% of the total number of nucleated cells in tendon tissues [20] and have been reported to differentiate into tenocytes, as well as along the chondrogenic, osteogenic and adipogenic lineages upon in vitro induction; and to originate tendon-, cartilage-, bone- and tendon-bone junction-like tissues in animal models [20,54,55,64,73]. Indeed, TDSCs have shown strong healing ability upon cell injection into a rat achilles tendon injury model [74]. These results highlight the possible contribution of tendon stem cell populations toward the generation of tendon-like tissue upon injury, but the mechanisms involved are still to be fully understood.

Nonetheless, the limited cell number obtained upon isolation from tendon tissues requires an additional step of in vitro expansion, which leads to phenotypic drift [75] and consequent reduction of healing capacity. Hence, recent technological advances propose the use of epigenomic approaches to maintain the tenogenic phenotype of TSPCs [76]. Indeed, the treatment of TSPCs with inhibitors of histone deacetylase activity enabled significant cell expansion without phenotypic alterations and treated TSPC sheets were able to accelerate tendon repair in an in-vivo rat model of pattelar tendon injury [76]. Although these outcomes open new avenues toward the potential application of tendon stem cells for regenerative therapies, additional steps of cellular manipulation in vitro make clinical translation more difficult.

#### 3.3.2. Mesenchymal Stem Cells from Non-Tendon Tissues

Bone marrow-derived MSCs are the most commonly explored source of stem cells in tendon tissue engineering and regeneration approaches [52,77,78,79,80]. Nonetheless, adipose tissue-derived stem cells are also being increasingly explored for enhancing tendon repair owing to the easier accessibility for tissue harvesting and higher proliferative capacity, which renders increased cell numbers for clinical application. Although studies with MSCs have been targeting tenogenic differentiation of these cell sources, it is most likely that the therapeutic effects will be exerted by the empowering capacity of stem cells over tendon resident cell populations. Both bone marrow aspirate and adipose tissue constitute interesting stem cell sources for the translation of bioengineering approaches to the bedside, holding strong potential for managing tendinopathies in the clinics. Various clinical studies are currently being performed to better understand the clinical potential of MSCs in treating tendinopathies (Table 2). Recent evidences of the role of these cell types in tendon tissue engineering and regeneration strategies are illustrated below.

• Bone marrow-derived MSCs

BM-MSCs are commonly harvested through a minimally invasive aspiration procedure from the iliac crest and then isolated from the mononuclear cell fraction upon density centrifugation. BM-MSCs have been frequently used as a comparison in tendon stem cell characterization studies and are known to express several tendon-related markers, including scleraxis, tenomodulin, collagen types I, II and III, decorin, biglycan, although to a lower extent than TSPCs [20].

In vitro co-culture studies have been demonstrating the role of bi-directional crosstalk between tendon cells and BM-MSCs on the induction of a possible tenogenic phenotype through an up-regulation of tendon-related genes, including scleraxis and tenomodulin, and tendon ECM markers, like collagen type I, decorin and tenascin, together with significant ECM deposition [52,80]. Overall, these studies have been suggesting a role of BM-MSCs in enhancing the tenogenic properties of TDSCs and TSPCs, mostly through increased deposition of collagenous proteins and the recreation of a tendon-like ECM, favoring tenogenic differentiation.

Interestingly, BM-MSCs have been reported to generate embryonic tendon-like tissue in vitro through a process that is mediated by TGF-β3 signaling and requires a 3D environment [81]. In this study, BM-MSCs were cultured in fixed-length fibrin gels and were able to spontaneously generate collagen fibrils identical to those of an embryonic tendon [81]. These results highlight the potential contribution of BM-MSCs to tendon tissue engineering strategies.

Furthermore, BM-MSCs are being explored for their role in supporting tendon cells, particularly through the release of paracrine factors. Pre-conditioning tendon cells by in-vitro culture with BM-MSCs secretome and further combining those cells with an electrospun keratin-based scaffold resulted in improved biomechanical performance in an in-vivo rat model of chronic massive rotator cuff tear [82]. These outcomes suggest a beneficial effect of BM-MSCs secretome, which has been demonstrated to include several growth factors, cytokines and other soluble molecules intervening in cellular growth and/or maintenance and signal transduction processes [83].

Besides in-vitro tendon cell priming, other bioengineering approaches include the resort to scaffold-free cell sheet engineering. Cell sheets derived from co-culturing TDSCs and BM-MSCs have been reported to support tendon healing in a rat patellar tendon window injury model [80]. The combination of both cell types outperforms the effects of each cell population individually. It has been demonstrated that the ratio between both cell types is a crucial aspect for tissue repair; a 1:1 cell ratio led to the best therapeutic effect upon cell sheet implantation, resulting in a higher number of elongated cells and better orientation of collagen fibers, as well as the best mechanical performance [80]. Indeed, co-culture approaches constitute remarkable tools in overcoming some challenges of cellular therapies for tendon applications. For instance, TDSCs alone exhibited better regenerative ability than BM-MSCs alone upon cell injection into a rat achilles tendon injury model [74], but the limited number of TDSCs obtained upon isolation hinders clinical translation of TDSCs as a source for cellular therapies, further supporting the combination of tendon cell populations with BM-MSCs

In summary, BM-MSCs are paving their way in tendon regenerative therapies owing to their contribution to new tendon-like tissue formation and to boost the tenogenic properties of tendon cells populations.

• Adipose tissue-derived MSCs (ASCs)

ASCs constitute an interesting candidate cell source for tissue engineering and regenerative medicine applications. These cells can be isolated from subcutaneous adipose tissue and, more recently, from liposuction aspirates [84,85] and have been reported to improve tendon healing in in-vivo tendon injury models [72,86]. Similarly to BM-MSCs, the mechanisms behind such therapeutic effects have not been elucidated so far, but attempts to understand the role of ASCs have also been exploiting co-culture systems with tendon resident cells. Comparably to BM-MSCs, co-culture studies demonstrated that the cellular crosstalk leads to an up-regulation of tendon-related genes [87,88]. Nonetheless, difficulties in distinguishing between both cell types limit the interpretation of these outcomes as to whether MSCs are differentiating along the tenogenic lineage or boosting the tenogenic properties of tendon cells. We have recently addressed the influence of ASCs over tendon niche using indirect (transwell) co-cultures with tendon explants. Interestingly, ASCs seemed to aid in preserving the architecture of native tendon tissue over time in culture [89]. Additionally, collagenolytic activity of matrix metalloproteinases (MMPs) was increased in co-cultures, suggesting a fastened ECM remodeling [89]. This was further investigated in direct contact co-culture systems using ASCs and human tendon-derived cells, demonstrating an accelerated deposition of ECM components, particularly collagen type I, and improved COL1/COL3 (collagen type I to collagen type III) ratio [88]. Given the role of collagen type III in fibrotic healing and scar tissue formation, these results suggest that ASCs may have a beneficial effect toward shifting the repair microenvironment. Not only were ASCs able to support tendon cells, but they were also spontaneously more elongated in co-culture systems, demonstrating a possible commitment toward the tenogenic lineage [88,89]. Further studies using cellular labeling techniques may help shed light over the biological events involved.

Additional concerns that must be undoubtedly considered arise from the hypocellular nature of tendon tissues. ASCs exhibit a high proliferation rate, whereas tendons possess relatively low cell numbers and an overproliferative phase may lead to fibrotic tissue formation and scarring. Strikingly, co-culturing ASCs with tendon-derived cells enabled a control over the proliferation rate; indeed, lower cell numbers have been reported for co-culture systems in comparison to ASCs cultured alone [88].

Altogether, these results support a potential therapeutic effect for ASCs as they have been able to modulate the microenvironment of tendon niche in vitro, as well as to improve tendon healing in vivo, but more studies at the molecular level will be useful to help clarify the exact mechanisms behind these responses.

## 4. Conclusions and Perspectives

Evidences from both in vitro and in vivo studies suggest that tendon resident cells, including tendon MSCs (TSPCs or TDSCs), may be the main orchestrators directing tendon-regenerative processes. Nonetheless, such biological response upon injury may be further boosted by the administration of non-tendon MSCs through two distinct but synergistic mechanisms—“cell replacement” and “cell empowerment”. Indeed, MSCs include cellular populations with potential therapeutic contributions by homing to the target site and exhibiting the ability to reconstitute/repopulate the injured tissue, simultaneously exerting an immunomodulatory effect that may shift the inflammatory environment. These events are crucial in switching tendon repair from pro-fibrotic to tissue regeneration, but functional clinical outcomes are still to be investigated.

Future research in the field of cellular therapies for tendon regeneration must address several issues that still pose a huge obstacle to clinical translation, including the lack of standardized methods and the inexistence of an optimal panel of markers for characterizing tenogenic differentiation steps.

Furthermore, there is clear influence from the bi-directional crosstalk between tendon MSCs and BM-MSCs or ASCs in enhancing the tenogenic properties of native cell populations. Nonetheless, the cellular and molecular mechanisms involved in these processes need to be investigated in more detail. Notwithstanding, tendon tissue engineering and regeneration can evolve from the combination of MSCs of different origins with biomaterials’ support to improve tendon healing and simultaneously provide adequate mechanical performance at the tissue level.

## Figures and Tables

**Table 1:** Protocols for tendon MSCs populations isolation and main characteristics.

Isolation Method	Tissue Origin	Cell Type	Method Description	Culture Medium	Characterization					Differentiation Potential	Ref.
					Morphology	Gene Expression	Protein Expression	Flow Cytometry			
**Animal Origin**											
Digestion	Mouse Patellar tendons	TSPCs	Removal of tendon sheath and surrounding paratenon; cut into small pieces; digested with 3 mg/mL collagenase type I + 4 mg/mL dispase/PBS (1 h, 37 °C)	α-MEM + 20% lot-selected FBS + 100 mM 2-mercaptoethanol	Heterogeneous colonies	*Scx*, cartilage oligomeric protein (*Comp*), *Sox9* and *Runx2*	Expression of collagen type I, fibronectin, COMP, tenascin-c Low expression of α-SMA	Positive Sca-1 CD90.2 CD44	Negative CD34 CD117 CD45 Flk-1 CD18 CD144	Osteogenic Adipogenic	[20]
Digestion	Rat Flexor Tendons	TDSCs	Removal of peritendinous connective tissue; tissue minced and digested with 3 mg/mL collagenase type I (2.5 h, 37 °C); strained through a 70 µm cell strainer; suspension washed in PBS (centrifuge 300× *g*, 5 min); cells resuspended in medium	DMEM + 10% FBS + 100 U/mL penicillin + 100 µg/mL streptomycin + 2 mM L-glutamine	P0: large polygonal and star-shaped cells; P1: flat and slender cells; P3: fibroblast-like cells; Heterogeneous colonies		Expression of α-SMA, tenascin-c, tenomodulin and aggrecan	Positive CD90 CD44	Negative CD34 CD31	Osteogenic Chondrogenic Adipogenic	[54]
Digestion	Rabbit Patellar and Achilles Tendons	TSCs and tenocytes	Tendon portions (1 mm^3^) minced; 100 mg of tissue sample digested with 3 mg collagenase type I + 4 mg dispase/1 mL of PBS (1 h, 37 °C); suspensions were centrifuged (1500× *g*, 15 min); cell pellet resuspended; tenocytes were obtained by application of local trypsin to colonies and culture in T25 flasks	DMEM + 20% FBS + 100 U/mL penicillin + 100 µg/mL streptomycin Tenocytes: DMEM + 10% FBS	TSCs: Cobblestone shape; unequal colonies formation Tenocytes: elongated shape	TSCs differentiated: Expression of *Runx2, Sox2* and *Col2A1*	Expression of Oct-4, SSEA-4 and nucleostemin in TSCs Tenocytes exhibited an absence for the same markers			TSCs: Osteogenic Chondrogenic Adipogenic	[55]
Digestion	Rat Patellar Tendons	TDSCs	Removal of peritendinous connective tissue; tissue was minced; digested with 3 mg/mL of collagenase type I; strained through a 70 µm cell strainer; cell suspension resuspended in culture medium	LG-DMEM + 10% FBS + 100 U/mL penicillin + 100 µg/mL streptomycin + 2 mM L-glutamine	Colonies formation	Expression of *Scx, Col1A1, Dcn, Alp, Col2A1, Col3A1, Tnc, Aca, Tnmd, Oct4, Sox2, Nanog*		Positive CD90 CD73	Negative CD45	Osteogenic Chondrogenic Adipogenic	[56]
Digestion	Rat Patellar Tendons (Diseased and healthy)	TDSCs	Removal of peritendinous connective tissue; tissue was minced; digested with 3 mg/mL of collagenase type I; strained through a 70 µm cell strainer; cell suspension resuspended in culture medium	LG-DMEM + 10% FBS + 100 U/mL penicillin + 100 µg/mL streptomycin + 2 mM L-glutamine	Colonies formation	Higher expression of *Col1A1, Scx* and *Tnmd* in healthy TDSCs	Expression of SOX9	Positive CD90 CD44* CD73* *Lower levels in diseased TDSCs	Negative CD31 CD34 CD45 CD11b	Osteogenic Chondrogenic Adipogenic	[57]
Digestion	Rat Achilles tendons	TSCs/TSCs Sheets	Removal of tendon sheath and paratenon; tissue minced into small pieces; about 100 mg of tissue digested with 3 mg/mL of collagenase type I and 4 mg/mL of dispase in PBS (2.5 h); centrifuged at 1500× *g*, for 15 min; cell suspension strained through a 70 µm cell strainer; cell pellet resuspended in medium in T25 flasks	DMEM + 10% FBS + 100 U/mL penicillin + 100 µg/mL streptomycin	Heterogeneous colonies; P3: homogeneous and cobblestone-shape cells		Expression of collagen type I, collagen type II and tenomodulin	Positive CD90 CD44	Negative CD31 CD34 CD45	Osteogenic Adipogenic	[58]
Digestion	Fetal Bovine Achilles Tendons	TDSCs	Removal of peritendinous connective tissue; washed with PBS; tissue trimmed into 1 mm^3^ pieces; digested with 0.1% collagenase type I (1 h, 37 °C); sample strained through a 70 µm cell strainer and added complete medium to stop reaction; cell suspension centrifuged (1200 rpm, 10 min); cells resuspended in culture medium	LG-DMEM + 15% FBS + 100 U/mL penicillin + 100 µg/mL streptomycin + 2.5 ng/mL bFGF and 2 mM L-glutamine	Spindle-shaped or fusoid cells; Colonies formation	Expression of *Col1A1, Tnc*, *Col3A1* and *Cd44*	Expression of Collagen type I and III, CD44 and Tenascin-C			Osteogenic Adipogenic Chondrogenic	[59]
Digestion	Rat Achilles Tendons	TDSCs	Removal of tendon sheath and paratenon; tissue cut into small pieces; digested with 2 mg/mL collagenase type I (2.5 h, 37 °C); resuspend in culture medium;	DMEM + 10% FBS + 50 µg/mL penicillin + 50 µg/mL streptomycin +100 µg/mL neomycin		Up-regulation of *CD90*, nucleostemin (*NS*), *Col3A1,* lysyl oxidase *(Lox), Tnc;* Low expression of *Tnmd*, *Dcn* and *Fmod*				Tenogenic	[60]
Digestion	Murine	TDSCs and tenocytes	Tissue digested for 3 h at 37 °C in 20 mL 375 U/mL collagenase type I and 0.05% trypsin; cell suspension strained and centrifuged at 1200× *g* for 10 min; cells resuspended in medium	DMEM + 20% FCS +100 U/mL penicillin + 100 μg/mL streptomycin and 2 μg/mL amphotericin B	TDCs: smaller and round shaped; Tenocytes: large and flat fibroblast-like cells	TDCs: *Nanog*, CD73, CD45, *Scx* and *Mkx*; Tenocytes: *Tnc*, thrombospondin-4 and *Tnmd*				Osteogenic Adipogenic	[61]
**Human Origin**											
Digestion	Hamstring tendons	TSPCs	Removal of tendon sheath and surrounding paratenon; cut into small pieces; digested with 3 mg/mL collagenase type I + 4 mg/mL dispase/PBS (1 h, 37 °C)	α-MEM + 20% lot-selected FBS + 100 mM 2-mercaptoethanol		Expression of *Tnmd*, *Comp*, *Runx2*		Positive Stro-1 CD146 CD90 CD44	Negative CD18 CD34 CD45 CD117 CD106	Osteogenic Adipogenic Chondrogenic	[20]
Explant	Hamstring Tendons	TDCs	Removal of peritendineum; cut into 3 mm^3^ pieces and placed in culture medium	DMEM + 10% FCS + 50 µg/mL gentamicin + 1.5 µg/mL fungizone	Spindle-shape			Positive CD105	Negative CD34	Osteogenic Adipogenic Chondrogenic	[62]
Digestion	Supraspinatus and long head of biceps tendons	TDSCs	Washed with PBS; tissue was cut into small pieces and digested with 3 mg/mL collagenase type I and 4 mg/mL dispase, in PBS (1.5 h, 37 °C); sample was centrifuged; cell pellet resuspended in culture medium	α-MEM + 20% lot-selected FBS + 2 mM L-glutamine + 100 U/mL penicillin + 100 µg/mL streptomycin	Fibroblast-like shape; Colonies formation	Expression of *Col1A1, Col3A1, Mmp-2, TGFB1, Cx43*		Positive CD90 CD44 CD146	Negative CD2 CD3 CD11b CD14 CD15 CD16 CD19 CD56 CD123 CD235a CD18 CD34 CD117	Osteogenic Adipogenic Myogenic	[63]
Digestion	Fetal Achilles Tendon	TSPC	Tissues were cut into 1–2 mm^3^ pieces and washed 3x with PBS; digested with 0.25% collagenase (37 °C, overnight); cell suspension was cultured in culture medium;	DMEM low glucose + 10% FBS + 1% penicillin-streptomycin	Fibroblast-like morphology			Positive CD105 CD90 CD146	Negative CD44 CD18 CD34	Osteogenic Chondrogenic Adipogenic	[64]
Digestion	Hamstring Tendons	TSC	Tissue was harvested in 8 mm^3^ blocks; surrounding adipose and muscle tissues were cleaned off; samples were cut into small pieces and digested with 3 mg/mL collagenase type I + 4 mg/mL dispase in PBS (1 h, 37 °C); cell suspension was centrifuged (2000 rpm, 15 min); cell pellet was resuspended in culture medium	α-MEM + 20% FBS + 1% penicillin and streptomycin + 100 mM 2-mercaptoethanol	Elongated fibroblastic-like cells and polygonal-shaped cells		Expression of CD146, STRO1, α-SMA and Tenomodulin	Positive CD44		Osteogenic Adipogenic Chondrogenic	[65]
Explants	Rotator cuff Tendons	TSPC	Tissue was minced into 1 mm^3^ pieces, placed on a 10-mm diameter culture dish and cultured in a monolayer with medium; minced tissue was removed after 1 week; after 2–3 weeks, the cells were harvested with 0.05% trypsin/EDTA and cultured in non-coated flasks	α-MEM + 10% heat-inactivated FBS + 2 mM L-glutamine + antibiotics	Fibroblast-like spindle shape;			Positive CD29 CD44 CD105 CD166	Negative CD14 CD34 CD45	Osteogenic Adipogenic Chondrogenic	[66]
Digestion	Gracilis and semitendinosus tendons	TSPCs within tendon resident cells	Tissue was fragmented and digested with 0.3 % (*w*/*v*) collagenase type I (16 h, 37 °C) in HG-DMEM; samples were strained through a 100 µm cell strainer and centrifuged (300× *g*, 5 min); cells were plated in culture medium;	HG-DMEM + 10% FBS 50 µg/mL penicillin + 50 µg/mL streptomycin +2 mM L-glutamine Cultured in the presence/absence of 5 ng/mL bFGF	Fibroblast-like shape; Low colony forming capability	Expression of *Scx*, *Tnc*, *Mkx*, *Oct4* (in presence and absence of bFGF)		Positive CD13 CD73 CD90 CD54	Negative CD34 CD45	Osteogenic Chondrogenic	[67]

Abbreviations: αMEM, Minimum Essential Medium alpha; αSMA, Alpha Smooth Muscle Actin; bFGF, basic Fibroblast Growth Factor; Cx, Connexin; DMEM, Dulbecco’s Modified Eagle’s Medium; EDTA, Ethylenediamine tetraacetic acid; FBS, Fetal Bovine Serum; FCS, Fetal Calf Serum; HG-DMEM, High Glucose DMEM; LG-DMEM, Low Glucose DMEM; Mkx, Mohawk; MMP, Matrix Metalloproteinase; PBS, Phosphate Buffer Saline; TGF, Transforming Growth Factor. Stro-1 was the first mesenchymal stem cell marker identified; Stro represents the stroma/mesenchyme.

**Table 2:** List of active clinical trials using MSCs to treat tendon injuries.

Cells	Condition	Strategy	Reference
**BM-MSCs**	Rotator cuff tears	Arthroscopic repair combined with bone marrow aspirate	NCT03688308
	Non-retracted supraspinatus tendon tear	Regenexx-SD injection: Bone marrow aspirate injection into the area of the damaged tendon	NCT01788683
	Full thickness rotator cuff tears	Arthroscopic repair combined with bone marrow aspirate	NCT02484950
**ASCs**	Intractable common extensor tendinosis	Intra-tendon injection of allogeneic ASCs combined with fibrin glue	NCT03449082
	Rotator cuff tear Lateral epicondylitis	Injection of autologous ASCs (1 million cells/10 kg body weight) once a week, three times	NCT03279796
	Partial-thickness rotator cuff tear Rotator cuff tendinitis	Single injection of adipose-derived regenerative cells (ADRCs)	NCT03752827

## Abbreviations

AFSCs	Amniotic fluid stem cells
ASCs	Adipose-derived stem cells
bFGF	Basic Fibroblast Growth Factor
BM-MSCs	Bone marrow-derived mesenchymal stem cells
BMP	Bone Morphogenetic Protein
ECM	Extracellular matrix
EGF	Endothelial growth factor
GBD	Global Burden of Disease
IGF	Insulin-like Growth Factor
IL	Interleukin
iPSCs	Induced pluripotent stem cells
MMPs	Matrix metalloproteinases
MSCs	Mesenchymal stem cells
PDGF	Platelet-derived Growth Factor
PRHd	Platelet-rich hemoderivatives
PRP	Platelet-rich plasma
TDSCs	Tendon-derived stem cells
TGF	Transforming Growth Factor
TSPCs	Tendon stem/progenitor cells
